# The Egyptian Sphynx

**Published:** 1883-09

**Authors:** 


### THE EGYPTIAN SPHYNX.

ON the western bank of the Nile, and near the second largest pyramid, stands this famous object, which has from time immemorial so excited the awe and curiosity of the traveler. As long ago as the time of Herodotus, the first man who ever conceived the idea of writing a history, and who lived four or five hundred years before the time of Christ, this gigantic and mysterious human head was raised above the sand, looking upon the beholder with the same calm and serene expression, as if superior to the vexations of life and to the mutations of time, as much a puzzle as it is in our own day. It was then hoary with age, though over two thousand years have since rolled away. First the Persians, under Cambyses, before Herodotus' time, invaded and devastated the country. Then the Greeks, under Alexander and the Ptolemys, his successors; then the Moslems, one of whom, as a hater of images, sought in vain to destroy it; and then many other lesser powers down to the Turks of the present day; all alike unaffected by any respect for the monuments of antiquity, and ignorant of their value as records of human progress and decay.

The deep mist and darkness of the Middle Ages rolled over it for centuries, and still the Sphynx remains one of the wonders of the ancient world, mutilated, disfigured, neglected and sometimes almost forgotten; but always serene, impressive and awe-inspiring.

It is hewn from the salient angle of the rocky ledge on which stand the great pyramids; part of the cap, the back and the fore-paws, when the rock was abraded, being supplied by masonry. The savants, under Napoleon, uncovered the back and shoulders, and obtained approximate measurements; but it was not until 1818 that the entire figure was displayed to its pedestal. The honor of this work is due to the Italian Caviglia. The loose sand, anciently restrained by artificial irrigation, and doubtless highly cultivated, but now continually shifting about in that rainless country with the slightest breath of air, was removed by the labor of from sixty to eighty persons for several days. No trace of these excavations, made at so much cost, now remains; the upper part of the figure alone being seen, rising as before about thirty or forty feet above the level of the soil. It faces eastward toward the Nile. At the rear is the pyramid of Shafra, while toward the north, in a corresponding position in reference to the pyramid, stands another rock, which, in the opinion of Lepsius, it may have been the intention of the builder to carve into another Sphynx, as a twin guardian of the approach to the sacred pile.

Caviglia's efforts laid bare a descent from the Nile for 135 feet succeeded by a flight of thirteen steps, after which was a platform and a second flight of thirty steps to the rocky pavement on which the figure rests. The body is that of a lion, with human head and shoulders. Though often spoken of as feminine in features, the broken fragments of a beard which have been found demonstrate the incorrectness of the assertion. Indeed, among thousands of sphynxes found in Egypt, but one is known having a female head though those of the Greeks are almost always male.

The posture is recumbent, with the forepaws stretched out in front a distance of fifty feet. The body is 143 feet long, and the height to the top of the head is 62 feet. Between the forepaws is a level space which was used as a temple or a place of sacrifice. Here were the remains of an altar with charred embers still preserved —the relics of a sacrifice offered, perhaps, to avert the tide of invasion that overwhelmed the worshipers. Several small sphynx-like figures stood near or under the chin. Under the chin is an immense granite tablet, fourteen feet high, seven broad and two thick, covered with hieroglyphics relating to one Thothmes IV. and flanked by two other tablets of limestone. These formed part of the inclosure of the sacred area.

The traveler, Dr. Pococke, asserts that there are openings in the back and in the top of the head; but these could have been nothing but seams in the rock or spaces left by dislodgment of parts of the masonry, as Lepsius, a most careful observer, who visited it in 1843, states that no opening to the interior whatever was to be seen, and that the mass of the statue obviously must have been hewn from the native rock.

The following striking passage as to its present appearance is from Denon :

"Though its proportions are colossal, its outline is pure and graceful and the expression mild, gracious and tranquil. The character is African, but the mouth, the lips of which are thick, has a softness and delicacy of execution truly admirable. That it is an Egyptian head is plainly evident, notwithstanding the mutilation ; but the type is fuller and broader than usual in Egyptian statues."—*Illustrated World.*

---

A BAG of hot sand is a good comforter for cold feet in winter, if a hot water bag is not at hand.

CREAM cures sunburn on some complexions, lemon juice on others and cold water suits still others best.

A MAN asked Mr. Moody, "Can't a man use tobacco and be a Christian? "Yes," answered Mr. Moody, emphatically, "a nasty one."—*Weekly Witness.*

